# Supplementary figures and images for: An approach to quantitate maternal transcripts localized in sea urchin egg cortex using RT-qPCR with accurate normalization
Source: PLoS One. 2022 Jun 16;17(6):e0260831. doi: 10.1371/journal.pone.0260831 (PMC9202947; doi:10.1371/journal.pone.0260831)

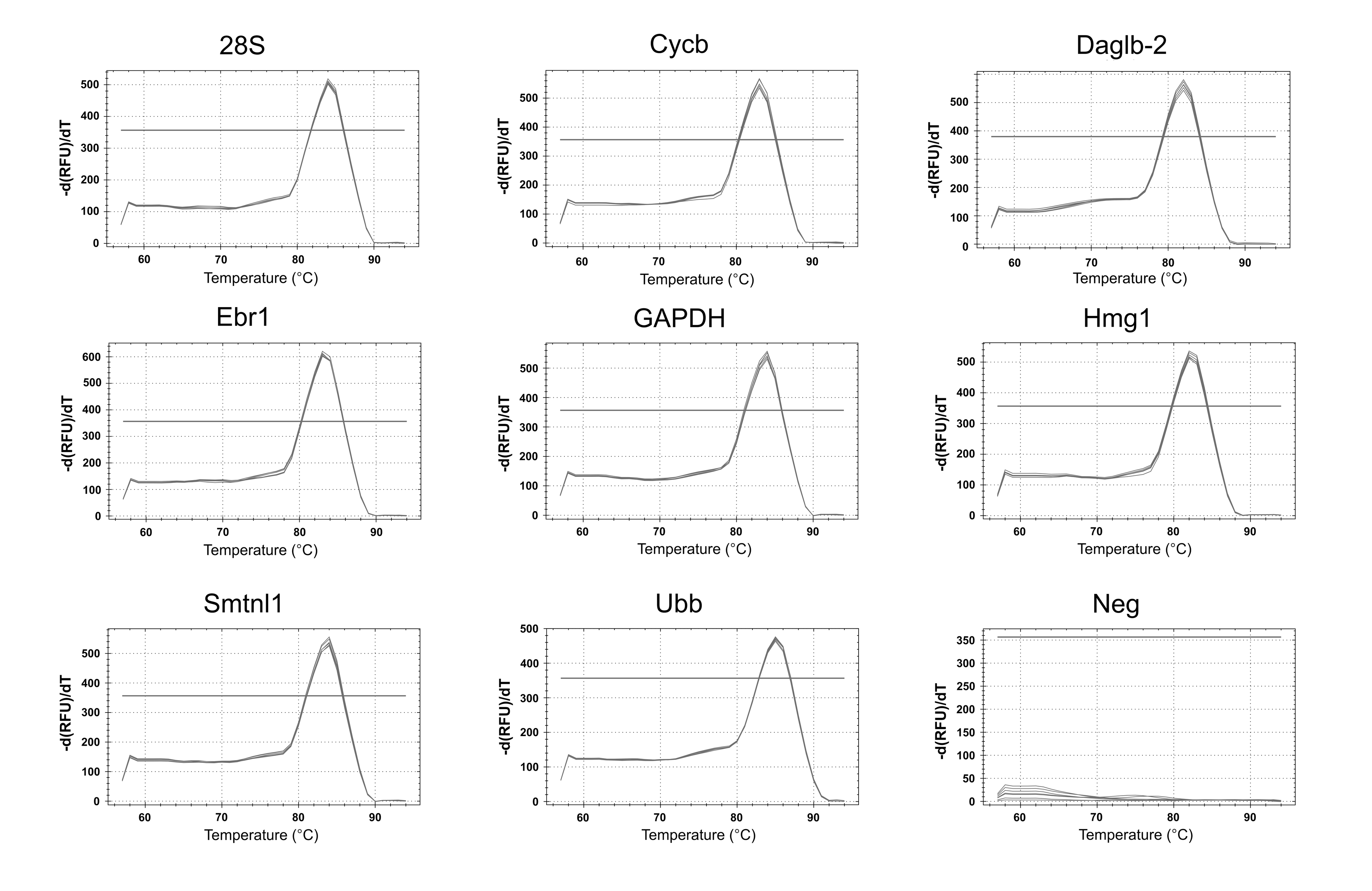

Supplement: S1 Fig — Egg and cortex melt curves are given together for each gene. Negative control (template-free) is marked by Neg. (TIF) [file pone.0260831.s001.tif]
